# Long-Term Asymmetrical Acceleration of Protein Evolution after Gene Duplication

**DOI:** 10.1093/gbe/evu159

**Published:** 2014-07-28

**Authors:** Oriol Pich i Roselló, Fyodor A. Kondrashov

**Affiliations:** ^1^Facultat de Medicina, Universitat de Barcelona (UB), Spain; ^2^Bioinformatics and Genomics Programme, Centre for Genomic Regulation (CRG), Barcelona, Spain; ^3^Universitat Pompeu Fabra (UPF), Barcelona, Spain; ^4^Institució Catalana de Recerca i Estudis Avançats (ICREA), Barcelona, Spain

**Keywords:** gene duplication, evolution, selection

## Abstract

Rapid divergence of gene copies after duplication is thought to determine the fate of the copies and evolution of novel protein functions. However, data on how long the gene copies continue to experience an elevated rate of evolution remain scarce. Standard theory of gene duplications based on some level of genetic redundancy of gene copies predicts that the period of accelerated evolution must end relatively quickly. Using a maximum-likelihood approach we estimate preduplication, initial postduplication, and recent postduplication rates of evolution that occurred in the mammalian lineage. We find that both gene copies experience a similar in magnitude acceleration in their rate of evolution. The copy located in the original genomic position typically returns to the preduplication rates of evolution in a short period of time. The burst of faster evolution of the copy that is located in a new genomic position typically lasts longer. Furthermore, the fast-evolving copies on average continue to evolve faster than the preduplication rates far longer than predicted by standard theory of gene duplications. We hypothesize that the prolonged elevated rates of evolution are determined by functional properties that were acquired during, or soon after, the gene duplication event.

## Introduction

The study of gene duplications continues to be at the forefront of molecular evolution due to their likely role in the emergence of new functions (Bridges 1936; Ohno 1970; Conant and Wolfe 2008; Ponting 2008; Hahn 2009; Innan and Kondrashov 2010). New functions are thought to emerge in the process of sequence and regulatory divergence of the gene copies that result from a gene duplication event. Therefore, the efforts in theory and empirical observations of gene duplications remain focused on the rate of evolution after gene duplication. Models of gene duplication evolution suggest that either one (Ohno 1970; Hughes 1999) or both (Force et al. 1999) gene copies experience a brief period of accelerated evolution (Hahn 2009; Innan and Kondrashov 2010), an elevated ratio of nonsynonymous (d*n*) and synonymous (d*s*) rates, followed by a return to the preduplication levels (Walsh 1995; Stoltzfus 1999; Lynch and Conery 2003; Konrad et al. 2011; Proulx 2012). It is thought that within the initial period of evolution new functions are forged.

Several approaches have been used to detect the acceleration of evolution after a gene duplication. First, d*n*/d*s* ratios between duplicated pairs in the same genome were correlated with d*s* values, where d*s* is a proxy for time since duplication. The observation that for values of d*s* > 0.1 the d*n*/d*s* appear to be relatively constant was taken as evidence that the observed plateau must correspond to the preduplication levels (Lynch and Conery 2000; Vinogradov 2012). Second, the d*n*/d*s* ratios of duplicated and nonduplicated genes consistently show that recently duplicated genes evolve faster than that of nonduplicated genes (Kondrashov et al. 2002; Nembaware et al. 2002; Jordan et al. 2004; Yampolsky and Bouzinier 2014), although genes that duplicated a long time ago appear to be more conserved (Davis and Petrov 2004; Jordan et al. 2004). The observation that d*n*/d*s* ratios measured in paralogous comparisons were higher than when the d*n*/d*s* is measured between nonduplicated orthologs suggested an acceleration of evolution in gene copies.

**Fig. 1.—:**
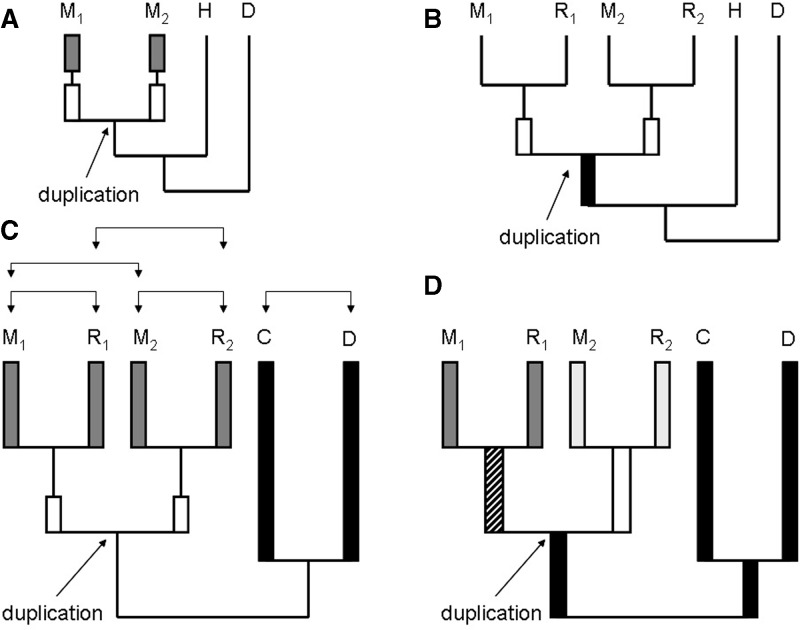
Phylogenetic topology of different approaches that may be used to study the evolution of gene duplications. When considering paralogs in only one species it is not possible to distinguish between recent evolution (gray box on the phylogeny) and the initial evolutionary acceleration (*A*). This issue can be resolved when considering d*n*/d*s* between orthologs of two species that have split after the gene duplication (*B*). However, if the preduplication rate of evolution is determined from the internal branch (black box) it may be difficult to resolve given the proximity to the expected acceleration of evolution immediately after the gene duplication (white boxes). In this study we avoid both of the compounding issues by focusing on five pairwise sequence comparisons, shown with brackets in (*C*). We can distinguish recent evolution of the duplicated genes (M_1_–R_1_ and M_2_–R_2_, gray boxes) from the initial acceleration occurring immediately after the gene duplication event (white boxes). Similarly, we use the divergence between sequences from two species (*C*, *D*) that have diverged prior to the gene duplication event as a proxy for preduplication rates of evolution (black boxes). We date the gene duplication by estimating d*s* between the two paralogs in the two species (M_1_–M_2_ and R_1_–R_2_). (*D*) We estimated d*n* and d*s* on the phylogeny while restricting the model to estimating five different d*n*/d*s* values across the following branch segments indicated as follows: Black = preD, striped = ipostD_1_, white = ipostD_2_, dark gray = rpostD_1_, light gray = rpostD_2_.

These two approaches, however, cannot be used to determine at which time point after the gene duplication event the gene duplications return to their preduplicated rates of evolution. The first approach suffers from a lack of a comparison to other genes, as it is not clear whether or not the plateau of d*s* > 0.1 (Lynch and Conery 2000) corresponds to the preduplication levels. The second approach compares different genes and as some genes are more likely to be duplicated than others (Kondrashov et al. 2002; Jordan et al. 2004; Kondrashov 2012) it may create an inherent bias. Furthermore, under both approaches the d*n*/d*s* ratio is measured between two paralogous sequences. Therefore, the d*n*/d*s* estimated across the time since the gene duplication and is expected to be elevated for all comparisons due to the inclusion of the initial fast period of evolution right after the gene duplication (fig. 1*A*). Thus, neither of these two approaches makes it possible to reliably address the issue of the long-term impact of gene duplication on the rate of evolution.

To measure the long-term effects of gene duplication on the rate of evolution, it is necessary to estimate the preduplication rate of evolution and the recent rate of evolution of the paralogs. For very recent gene duplications measuring the rate of recent evolution is trivial as an estimate of d*n*/d*s* between them is sufficient. It is feasible to estimate recent d*n*/d*s* values from an older gene duplication by comparing orthologs of the gene copies of two species that separated after the emergence of the gene duplication in question (fig. 1*B*).

The preduplication rate of evolution can be estimated as the d*n*/d*s* observed on the internal branch leading up to the gene duplication event (fig. 1*B*). This approach has been utilized by Pegueroles et al. (2013) and Cusack and Wolfe (2007). Briefly, Pegueroles et al. (2013) identified instances of gene duplication that were broadly characterized by a phylogenetic relationship in figure 1*B*. They studied whether or not the rates of duplicated genes measured as d*n*/d*s* between the mouse and rat orthologs of the duplicated genes were significantly different from the rate of evolution on the internal branch leading up to the gene duplication. They found that the younger gene duplications indeed evolve faster than the preduplication rates. However, the older gene duplications, those that occurred between approximately 70 and approximately 43 Ma, were found to evolve at a rate indistinguishable from that in the preduplication branch. Cusack and Wolfe (2007) utilized a congruent approach to study the long-term impact on the rate of evolution stemming from the whole-genome duplication (WGD) in yeast. The rate of preduplication evolution was similarly inferred from an internal branch leading up to the duplication event.

These two studies presented with slightly contradictory results. Pegueroles et al. (2013) suggested that gene duplications return to their preduplication rates of evolution relatively quickly, on the order of old world and new world primate divergence (Chatterjee et al. 2009). In contrast, Cusack and Wolfe (2007) observed that in yeast the surviving duplications from the WGD event continue to evolve faster than those genes that have lost their extra copy. Unfortunately, the Cusack and Wolfe (2007) study was focused on the remnants of the WGD event and used very long distances between some of the genes in the phylogeny, with d*s* > 1, reducing the reliability of the d*n*/d*s* estimates. An earlier study suggested that d*n*/d*s* on internal branches just prior to duplication may be accelerated in vertebrates (Johnston et al. 2007).

Here, we utilize two different methods to study the persistence of the acceleration of the rate of evolution after gene duplication. First, we employ a similar method to that used by previous studies (Cusack and Wolfe 2007; Johnston et al. 2007; Pegueroles et al. 2013) estimating the rate of evolution across different segments of the phylogeny that includes a recent gene duplication. Second, we investigate the rates of evolution after gene duplications using data from gene duplications with tangible synonymous divergence distance and avoiding estimating the rate of evolution on internal phylogenetic branches.

## Materials and Methods

We obtained protein-coding sequences for the mouse, rat, human, orangutan, dog, and cow from ENSEMBL (Vilella et al. 2009). We used the data in ENSEMBL to identify orthology relationships in these genomes. We selected the one-to-one dog–cow orthologs and for these orthologous pairs we identified instances of one-to-many orthology between dog and mouse (human) genes. For such mouse (human) genes, we selected those that showed a one-to-one orthology to the rat (orangutan) genes. This approach allowed us to identify a preliminary set of genes that were duplicated after the dog−human split but before the mouse–rat (human–orangutan) divergence, with the exception of those cases where a deletion of one copy occurred in the dog–cow lineage. Furthermore, these homologs included all cases when more than one duplication occurred along the aforementioned phylogenetic segment.

For all of these cases, we aligned all of the homologs using the protein sequence with MUSCLE (Edgar 2004) and reverse-translated to create a multiple nucleotide alignment. We then used the codeml package (Yang 2007) to estimate d*n*, d*s*, and d*n*/d*s* values across the phylogeny specifying five different areas of the phylogeny with independent d*n*/d*s* values with model = 2, clock = 0 and a user-defined tree that as shown in figure 1*D*. We also used the codeml program from the PAML package (Yang 2007) to estimate d*n* and d*s* between some pairwise comparisons of sequences from the sextuplet alignment.

For those homologous clusters that included more than one paralog in the mouse (human) genome, we estimated the order in which the duplications occurred. We obtained d*s* measurements for all pairwise mouse (human) paralogs. We then identified the pair with the smallest d*s* value as being the most recent gene duplication and created a set (((Mouse_1_–Rat_1_),(Mouse_2_–Rat_2_)),(Cow,Dog)) for that pair. We then removed at random one of the two paralogs and its rat (orangutan) ortholog and found the pair with the lowest d*s* among the remaining paralogs and the same set of six homologs was created. The procedure was then repeated recursively until only a single pair of mouse (human) paralogs was left. In the final data set, 25 out of 90 sets of six homologs originated from clusters representing more than one duplication between the dog–human common ancestor and the mouse–rat (human–orangutan) divergence.

To eliminate instances when the one-to-many orthology between dog and mouse (human) was caused by a gene loss in the dog–cow lineage, we eliminated all instances when the absolute d*s* values among the paralogous comparisons were higher than expected to have originated after the dog–human split. Specifically, we eliminated all instances when either of the pairwise comparisons shown in figure 1 had a d*s* > 1. This approach must have also concurrently eliminated most instances of misalignment or erroneous ortholog assignment. We estimated δ by subtracting d*n*/d*s* of the dog–cow orthologs from the d*n*/d*s* value of each of the two mouse–rat (human–orangutan) orthologous comparisons.

Synteny analysis was performed by corresponding in ENSEMBL the neighboring genes of each dog gene to the neighboring genes of the paralogs in the mouse (human) genome. A mouse (human) homolog was identified as the original gene copy if its gene neighbors were orthologs to the gene neighbors of the dog gene. We excluded tandem duplications from this analysis.

## Results and Discussion

We used mammalian genomes due to the availability of sequence data and an established phylogeny among major groups. We searched for gene duplications that have occurred after the split of Laurasiatheria (the group includes cow and dog) and Euarchontoglires (human and mouse) lineages but before the divergence of mouse–rat or human–orangutan lineages. Thus, we identified genes that were found in single copy in dog and cow, but were duplicated in mouse (human) and rat (orangutan) genomes (see Materials and Methods). Overall, we found 90 cases of such sextuplet homologs, with a gene duplication common to the mouse–rat (human–orangutan) lineage and single copy orthologs present in both the dog and the cow. We use a shorthand notation to label the cow (C), dog (D), mouse copy 1 (M_1_), mouse copy 2 (M_2_), rat copy 1 (R_1_), rat copy 2 (R_2_), human copy 1 (H_1_), human copy 2 (H_2_), orangutan copy 1 (O_1_), and orangutan copy 2 (O_2_) where M_1_–R_1_ (H_1_–O_1_) and M_2_–R_2_ (H_2_–O_2_) are one-to-one orthologs.

**Fig. 2.—:**
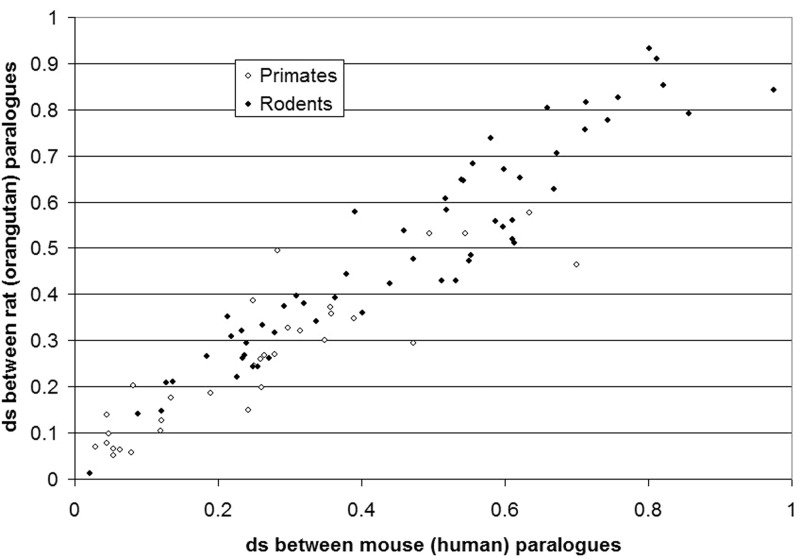
Relationship of synonymous divergence between gene duplications in sister species. The relationship between the synonymous divergence of paralogs in two species, M_1_–M_2_ (H_1_–H_2_) and R_1_–R_2_ (O_1_–O_2_) on the *Y* axis. The rodent and primate comparisons are shown with black and white circles, respectively.

Due to the possible prevalence of gene conversion we first analyzed whether or not different rates of synonymous divergence are observed between gene copies different species. We correlated the estimated d*s* values obtained from M_1_–M_2_ (H_1_–H_2_) and R_1_–R_2_ (O_1_–O_2_) pairwise paralogous comparisons. We observe a very good correlation of the d*s* in paralogs of different species (fig. 2), indicating that gene conversion was not a large factor in the evolution of these genes. Of course, we cannot exclude the possibility of frequent gene conversion that occurred in both mouse (human) and rat (orangutan) lineages for a specific gene duplication. However, we are certain that this cannot affect much of our data as in that case we would have expected to see more paralogous sequence comparisons with a low d*s*.

Next, we introduce, δ, a measure of the difference between the preduplication and the recent postduplication rates of evolution. We measured these rates of evolution in two different ways. In a maximum-likelihood approach, we used PAML (see Materials and Methods) to estimate the preduplication (preD), two initial postduplication (ipostD_1_ and ipostD_2_), and two different recent postduplication (rpostD_1_ and rpostD_2_) rates of evolution or d*n*/d*s* (fig. 1*D*). We estimated the d*n*/d*s* separately in the two different copies (fig. 1) due to the possibility of asymmetrical evolution of the gene copies (Zhang et al. 2003; Cusack and Wolfe 2007; Han et al. 2009; Panchin et al. 2010; Pegueroles et al. 2013) and classify the gene copies into those with high and low d*n*/d*s* values, fast and slow rate evolution, respectively. We then measured δ_f_ = max(rpostD_1_, rpostD_2_) − preD and δ_s_ = min(rpostD_1_, rpostD_2_) − preD, which represent a difference in the pre- and postduplication rate of evolution for fast- and slow-evolving gene copies, respectively.

**Fig. 3.—:**
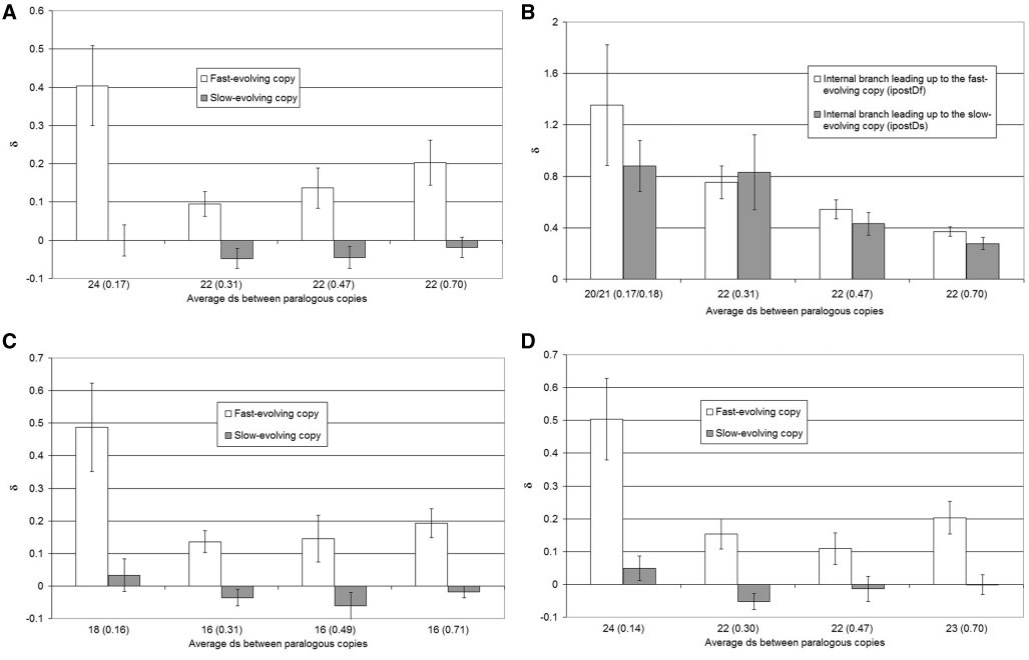
Difference in the rate of evolution in duplicated and nonduplicated orthologs. Average δ_f_ (white) and δ_s_ (gray) in bins. The number of duplications (average d*s* from the sum of branches connecting M_1_–M_2_ [H_1_–H_2_] and R_1_–R_2_ [O_1_–O_2_] nodes) is shown for each bin (*A*). The δ for internal branches (ipostD_f_ and ipostD_s_) is shown in (*B*), branches of length 0 were removed from the analysis resulting in 20 (average d*s* = 0.17) and 21 (average d*s* = 0.18) duplications in one bin for ipostD_f_ and ipostD_s_, respectively. The average δ_f_ (white) and δ_s_ (gray) excluding gene families are shown in (*C*). The average δ_f_ (white) and δ_s_ (gray) from pairwise comparisons are shown in (*D*), with average d*s* calculated similarly from pairwise comparisons of M_1_–M_2_ (H_1_–H_2_) and R_1_–R_2_ (O_1_–O_2_).

We used the average d*s* summed across the branches separating the paralogous gene copies M_1_–M_2_ and R_1_–R_2_ (H_1_–H_2_ and O_1_–O_2_; see fig. 1*D*), as a proxy for the length of time since the origin of the gene duplication. This allowed us to access how long after the gene duplication event the rate of evolution (d*n*/d*s*) remains elevated. We found three patterns when we compared δ_f_ and δ_s_ in duplications of different age (fig. 3*A*). First, the slower-evolving gene copies appear to evolve at the preduplication rates regardless of how long ago the duplication event occurred. This observation is consistent with our selection of gene duplications that are older than the mouse–rat divergence such that we were expected to miss the initial phase of acceleration that may be affecting the slow copy. Second, the rate of evolution in the younger fast-evolving duplications appears to be higher than in older duplications. Finally, the rate of evolution in fast-evolving gene duplications appears to be elevated even for the oldest duplications in our dataset with δ_f_ appearing to have reached a plateau at δ_f_ ∼ 0.15 for gene duplications older than d*s* ∼ 0.2 (fig. 3*A*).

When considering perfectly symmetrical rates of evolution one copy is expected to evolve slower than the other due to the random variation of the d*n*/d*s* measurements. However, in a truly symmetrical case the increase in the rate of evolution in the fast-evolving copy will be of the same magnitude as the decrease in the rate of evolution of the slow copy. Our observation that δ_f_ > 0 cannot be explained by the stochastic segregation of slow and fast gene copies because the observed increase of d*n*/d*s* in the fast copy is of much higher than the slight, insignificant decrease in the d*n*/d*s* of the slow copy.

The observation that the slow copy is evolving at the preduplication rate may be explained in two ways. First, the slow copy may have never experienced an acceleration in the rate of evolution. Second, the acceleration of evolution occurred but was short-lived such that it returns to normal within a modest timeframe (d*s* < 0.2). We therefore compared the rate of evolution of the initial postduplication branches leading up to the mouse–rat divergence (fig. 1*D*). We calculated δ_if_ and δ_is_ for the internal branch leading up to the slow copy and fast copy, respectively. Specifically, δ_if_ = ipostD_f_ − preD, where ipostD_f_ is the d*n*/d*s* value from the branch leading up to the branch with δ_f_. Conversely, δ_is_ = ipostDs − preD, where ipostD_f_ is the d*n*/d*s* value from the branch leading up to the branch with δ_s_. We found that δ_is_ and δ_if_ are not significantly different even for gene duplications that have emerged recently (fig. 3*B*), indicating that the slow copy evolves faster immediately after gene duplication and subsequently returns to the preduplication levels.

It has been suggested that the old copy, the one that is not relocated in a gene duplication event, typically evolves slower than the new copy after the gene duplication (Cusack and Wolfe 2007; Han et al. 2009). We see the same pattern in our data. Due to the prevalence of tandem duplications in mammals it was often not possible to distinguish between the old and the new copies. For 26 cases, however, we used data on synteny between the rodent (primate) genome and that of dog and cow genomes to discriminate between the original and the new copy. For 21 out of 26 duplications, the fast-evolving copy was the novel copy (Fisher’s exact test, *P* = 0.02). We hypothesize that the old copy is more likely to maintain the previous function and, therefore, more likely to have the preduplication levels of d*n*/d*s*.

Due to the decrease in selection pressure in primates caused by a smaller effective population size (Li 1997), it may be possible that the observed acceleration of the d*n*/d*s* in the duplicated genes is mostly influenced by the data from primate evolution. However, the fast-evolving gene copies are still significantly faster than the preduplication levels when we consider only rodent data, although the primate fast copies appear to have been accelerated to a greater degree (table 1). An increase in the strength of selection is anticipated in the rodent lineage relative to the cow–dog lineage (Li 1997), indicating that our observation of an increased δ_f_ is robust to lineage-specific changes in selection pressures.

**Table 1:** Average d*n*/d*s* Values (Standard Error) for Slow and Fast-Evolving Gene Copies in Rodents and Primates

	Fast Copy	Slow Copy
Rodents	0.13 (±0.027)	−0.026 (±0.015)
Primates	0.46 (±0.15)	0.038 (±0.06)

In our data set, we include genes that have duplicated more than once since the divergence of rodents (primates) and the dog–cow common ancestor. We employ a scheme to separate such instances into individual duplication events (see Materials and Methods); however, it may be possible that some of the gene copies in our data set are influenced by more than one recently emerged copy. We therefore performed the same analyses having retained only those genes that have experience only a single gene duplication event since the rodent (primate) and dog–cow divergence. We find the same patterns in the subset of genes with a single gene duplication (fig. 3*C*), indicating that multiple gene duplications do not substantially affect our results.

Finally, we have sought to replicate some of our results without attempting to reconstruct the rate of evolution in internal branches. We thus devised an approach that was based on five sequence comparisons among the orthologous and paralogous pairs of sequences estimating d*n* and d*s* (fig. 1*C*). We measured the rate of preduplication evolution *O* = d*n*/d*s* observed in the C–D comparison. We then measured *P*_1_ and P_2_ as d*n*/d*s* values in the M_1_–R_1_ (H_1_–O_1_) and M_2_–R_2_ (H_2_–O_2_) orthologous comparisons, respectively, which estimate the recent rate of postduplication evolution. We then measured δ_f_ = max(P_1_, P_2_) − O and δ_s_ = min(P_1_, P_2_) − O, which represent a difference in the pre- and postduplication rate of evolution for fast- and slow-evolving gene copies, respectively. The average d*s* of paralogous comparisons, M_1_–M_2_ and R_1_–R_2_ (H_1_–H_2_ and O_1_–O_2_) was used as a proxy for the length of time since the origin of the gene duplication (fig. 1*C*).

The data obtained through pairwise sequence comparisons confirm our conclusions that 1) the slower-evolving gene copies appear to evolve at the preduplication rates regardless of how long ago the duplication event occurred, 2) the rate of evolution is higher in younger fast-evolving duplications, and 3) the rate of evolution in fast-evolving gene duplications appears to be elevated even for the oldest duplications (fig. 3*D*). Due to the limitations of the pairwise comparisons using this approach we cannot confirm the observation of the initial acceleration in the rate of evolution of the slow-evolving gene copies.

## Conclusions

Our results are consistent with the observations that asymmetrical evolution in gene duplication is common (Zhang et al. 2003; Cusack and Wolfe 2007; Han et al. 2009; Panchin et al. 2010; Pegueroles et al. 2013; Yampolsky and Bouzinier 2014) and with persistent, long-term faster evolution after a gene duplication in yeast (Scannell and Wolfe 2008). The observed persistence in the acceleration of evolution is surprising as it is not predicted by the more widespread theories of gene duplication. Most models of gene duplications that are based on a certain level of redundancy of gene duplications predict that the effect of the gene duplication event will be eliminated by accumulating mutations relatively quickly (Walsh 1995; Stoltzfus 1999; Lynch and Conery 2003; Konrad et al. 2011; Proulx 2012). Our data suggest that one of the gene copies seems to acquire some property other than redundancy, such as a new function (Notebaart et al. 2005; Assis and Bachtrog 2013), either at the duplication event or later, that results in a persistent faster rate of evolution than the ancestral single copy gene, or its ancestrally positioned paralog.

The following view on gene duplication evolution emerges from our data. First, immediately after gene duplication both gene copies experience an initial acceleration in their rate of evolution. Subsequently, one of the gene copies, which is typically the copy in the original genomic location, quickly returns to the preduplication levels in the timeframe of d*s* ∼ 0.15. The second copy in the novel location eventually (d*s* > 0.2) achieves a stationary evolutionary rate. However, it does not appear to reach the preduplication levels of evolution even after a substantial period of time (d*s* > 0.6). Furthermore, the novel copy takes longer to reach the new stationary rate of evolution than is necessary for the old copy to return to the preduplication rates. Unfortunately, due to the scarcity of our data we cannot address the issue of deviation of specific gene copies from the described course of evolution. We believe that the functional characterization of individual cases may reveal the properties of the new gene copies that result in their persistent accelerated evolution.
